# The Effects of Introducing a Mobile App–Based Procedural Logbook on Trainee Compliance to a Central Venous Catheter Insertion Accreditation Program: Before-and-After Study

**DOI:** 10.2196/35199

**Published:** 2022-03-07

**Authors:** Robert Tamblyn, Jorge Brieva, Madeleine Cain, F Eduardo Martinez

**Affiliations:** 1 Division of Critical Care John Hunter Hospital Hunter New England Local Health District Newcastle Australia; 2 School of Medicine and Public Health Faculty of Health and Medicine University of Newcastle Newcastle Australia

**Keywords:** logbook, education, training, central venous catheter, CVC, intensive care, smartphone, mobile phone, mobile apps, mHealth, mobile health, accreditation program, digital health, digital record

## Abstract

**Background:**

To reduce complications associated with central venous catheter (CVC) insertions, local accreditation programs using a supervised procedural logbook are essential. To increase compliance with such a logbook, a mobile app could provide the ideal platform for training doctors in an adult intensive care unit (ICU).

**Objective:**

The aim of this paper was to compare trainee compliance with the completion of a logbook as part of a CVC insertion accreditation program, before and after the introduction of an app-based logbook.

**Methods:**

This is a retrospective observational study of logbook data, before and after the introduction of a purpose-built, app-based, electronic logbook to complement an existing paper-based logbook. Carried out over a 2-year period in the adult ICU of the John Hunter Hospital, Newcastle, NSW, Australia, the participants were ICU trainee medical officers completing a CVC insertion accreditation program. The primary outcome was the proportion of all CVC insertions documented in the patients’ electronic medical records appearing as logbook entries. To assess logbook entry quality, we measured and compared the proportion of logbook entries that were approved by a supervisor and contained a supervisor’s signature for the before and after periods. We also analyzed trainee participation before and after the intervention by comparing the total number of active logbook users, and the proportion of first-time users who logged 3 or more CVC insertions.

**Results:**

Of the 2987 CVC insertions documented in the electronic medical records between April 7, 2019, and April 6, 2021, 2161 (72%) were included and separated into cohorts before and after the app’s introduction. Following the introduction of the app-based logbook, the percentage of CVC insertions appearing as logbook entries increased from 3.6% (38/1059) to 20.5% (226/1102; *P*<.001). There was no difference in the proportion of supervisor-approved entries containing a supervisor’s signature before and after the introduction of the app, with 76.3% (29/38) and 83.2% (188/226), respectively (*P*=.31). After the introduction of the app, there was an increase in the percentage of active logbook users from 15.3% (13/85) to 62.8% (54/86; *P*<.001). Adherence to one’s logbook was similar in both groups with 60% (6/10) of first-time users in the before group and 79.5% (31/39) in the after group going on to log at least 3 or more CVCs during their time working in ICU.

**Conclusions:**

The addition of an electronic app-based logbook to a preexisting paper-based logbook was associated with a higher rate of logbook compliance in trainee doctors undertaking an accreditation program for CVC insertion in an adult ICU. There was a large increase in logbook use observed without a reduction in the quality of logbook entries. The overall trainee participation also improved with an observed increase in active logbook users and no reduction in the average number of entries per user following the introduction of the app. Further studies on app-based logbooks for ICU procedural accreditation programs are warranted.

## Introduction

### Background

Central venous catheter (CVC) insertion is a common procedure performed in the intensive care unit (ICU) with up to 78% of ICU patients requiring insertion of a CVC during their admission [1]. CVC insertion complications occur frequently and can be serious. Mechanical complications include arterial injury, hematoma, and pneumothorax, while delayed complications can include thrombosis and catheter-related bloodstream infections [2]. Mechanical complications have been reported in 2.1% of all subclavian and 1.4% of all internal jugular CVC insertions [3].

In teaching hospitals, trainees perform a large portion of this aspect of care. In a 2017 UK multicenter audit, 62% of all CVC insertions were performed by trainees [4]. This is important to note because complications are more common in less experienced hands [5]. Training and accreditation programs help to reduce the rate of both mechanical complications and catheter-related bloodstream infections associated with less experienced operators [6,7]. While theoretical knowledge can be attained via educational modules, junior doctors need adequate experience and exposure to achieve procedural competency [8].

Logbooks are used to facilitate training and supervision across many fields of postgraduate medicine. In Australia and New Zealand, logbooks are a mandatory requirement of the Royal Australasian College of Surgeons, the Royal Australasian College of Physicians, and the Australasian College for Emergency Medicine [9-11]. Some evidence-based guidelines, such as those published in the British Journal of Anaesthesia, strongly recommend the use of logbooks in the training of CVC insertion and maintenance [12]. Logbooks help clarify learning objectives and can facilitate communication between the trainee and their clinical supervisor. They encourage procedural supervision of trainees and help supervisors know when a trainee doctor is competent at performing a procedure independently [13].

Paper-based logbooks have historically had poor compliance as well as incomplete and invalid data entry [14]. On the other hand, electronic logbooks have shown benefits including more efficient data access and an increased ability for supervisors to monitor trainees [15]. In many parts of the world, electronic logbooks have replaced paper-based logbooks, with desktop and website applications being used as their user interface [16,17]. Despite its advantages, a computer-based platform can have inconveniences of its own, with immediate accessibility not always available to the logbook user and their supervisor. In recent years, smartphones have become ubiquitous in hospitals, with medicine-related mobile phone apps being used daily for education and training and as a clinical aid [18]. It is possible that a smartphone app could provide a convenient platform for an electronic logbook for ICU-based procedures such as CVC insertion.

### Objectives

This study aims to determine if in a tertiary-level ICU, the introduction of an app-based logbook, when compared to a paper-based logbook, was associated with an increase in trainee compliance with the logbook component of a CVC accreditation program.

## Methods

### Design

This is a single-center, before-and-after, retrospective observational study of prospective data of logbook compliance.

### Setting and Participants

This study was conducted in the John Hunter Hospital ICU between April 7, 2019, and April 6, 2021. The John Hunter Hospital ICU is a 27-bed mixed medical and surgical unit in a tertiary, university-affiliated teaching hospital. It has over 2000 admissions per year and has an average of approximately 1100 CVC insertions annually.

The John Hunter Hospital is accredited for ICU general, as well as cardiothoracic surgery, neurosurgery, and trauma ICU training by the College of Intensive Care Medicine of Australia and New Zealand. Its junior medical roster accommodates 34 junior doctors at different levels of training at any one time, who are completing 3-month, 6-month, or 12-month rotations as part of their postgraduate training.

The ICU established a formal accreditation program for CVC insertion in 2013, introduced to improve patient safety and reduce complication rates. In 2017, the program incorporated a mandatory web-based training module followed by a practical component.

To be accredited as an independent operator for a specific type of procedure, trainees need to be supervised completing a prescribed number of CVC insertions. Each insertion must be logged by trainees in a communal, paper-based logbook. Each entry must contain a minimum level of information and be signed by a valid supervisor. A major limitation of the paper-based logbook has been its poor accessibility to both trainees and supervisors, contributing to poor trainee compliance.

### Intervention

In April 2020, a smart phone app was developed using AppSheet, a low code mobile app builder [19]. The app was introduced to the John Hunter Hospital ICU to increase the use of the procedural logbook, previously limited to a paper-based logbook kept in a communal folder. The app combined a read-only daily unit roster with an interactive, updatable procedural logbook.

Interface design focused on rapid data entry, minimum text requirements, and field-level automation. Individuals would enter de-identified patient data regarding the CVC insertion procedure using their mobile device, and the data would be immediately uploaded to a secure, central, web-based database. The app could be installed on both iPhone and Android operating systems. The app was made available to all doctors working in the unit from April 6, 2020, following a brief introductory session. The session was repeated each 3-month term for all new trainees rotating through the unit. The paper-based logbook remained available to all trainees, and they could use either the paper-based or app-based logbooks to create new entries.

### Variables

The primary outcome was compliance with the CVC insertion logbook, as indicated by the number of logbook entries made by trainees as a proportion of the total number of CVC insertions documented in the patients’ electronic medical record (EMR) before and after the introduction of the app-based logbook.

The secondary outcomes were as follows: (1) to assess the quality of the information entered in the logbook by measuring the number of supervisor-approved logbook entries, evidenced by having a supervisor’s signature; (2) to assess trainee participation by comparing the number of active logbook book users in the before and after periods; and (3) to assess trainee adherence by measuring the proportion of first-time logbook users who logged 3 or more entries.

### Data Collection

Data were collected from the EMR for all standard CVCs, hemodialysis catheters, and peripherally inserted central catheters inserted in the unit from April 7, 2019, until April 6, 2021. The data collected included the following: type of line inserted, insertion time, ICU admission time, patient’s medical record number, and insertion site.

Our inclusion criteria were the following: (1) CVCs (standard central lines, hemodialysis catheters, and peripherally inserted central catheter lines); (2) inserted within the ICU; (3) in adult patients.

Our exclusion criteria were the following: (1) invasive lines other than a CVC (eg, arterial line, pulmonary artery catheter, extracorporeal membrane oxygenation catheter, and rapid infusion lines); (2) CVCs inserted in patients younger than 18 years old; and (3) any CVC inserted outside of the ICU (eg, emergency department, operating theatres, wards, and prehospital).

Each CVC insertion documented in the EMR that met the inclusion criteria and had none of the exclusion criteria was recorded. The paper-based and app-based logbooks were then interrogated looking for the relevant information about the CVC insertion each logbook entry was meant to correspond with. The data points of the EMR and logbook entries that had to match were as follows: (1) patient’s medical record number, (2) line type, (3) insertion site, and (4) date of insertion. Only if all these variables were the same for both the EMR and the logbook was the CVC insertion defined as having a corresponding logbook entry.

For each insertion having a corresponding logbook entry, we recorded additional data obtained from the logbook, including the proceduralist’s name, the presence of a supervisor's signature, and in which logbook, paper, or app did the entry appeared. If the same entry appeared in both paper and digital logbooks, its source was recorded as paper. Using hospital records, we determined variables about the proceduralist including their level of training and the amount of time working at John Hunter Hospital ICU. A first-time logbook user was defined as any trainee making a logbook entry in their first term working in the John Hunter Hospital ICU.

All data, including those of the patients’ and the trainees’ who performed the procedure, were de-identified and entered in a purpose-built, password-protected, electronic database. Study numbers were assigned to patients and trainees to minimize the risk of de-identification.

We analyzed the database to determine the number of CVC insertions in EMR having a corresponding logbook entry in the before and after period, the presence of a supervisor’s signature, and the details of the trainee who performed the insertion.

### Study Size

For study size, 365 days before and after the introduction of the app were included to allow the equal representation of all parts of the training year.

### Data Analysis

Descriptive statistics are reported as fractions and percentages. Chi-square and Fisher exact tests were used to compare categorical variables. A 2-sided *P* value of <.05 was considered statistically significant.

### Ethics

The Hunter New England Human Research Ethics Committee approved an exemption for ethical review for the study given its negligible risk (authorization AU202108-07).

Permission to access the paper logbook was granted by the director of the unit as part of a quality assurance. The trainees provided implied consent when installing the app for all data entered to be collected and analyzed.

## Results

### Data Collection

**Figure 1:**
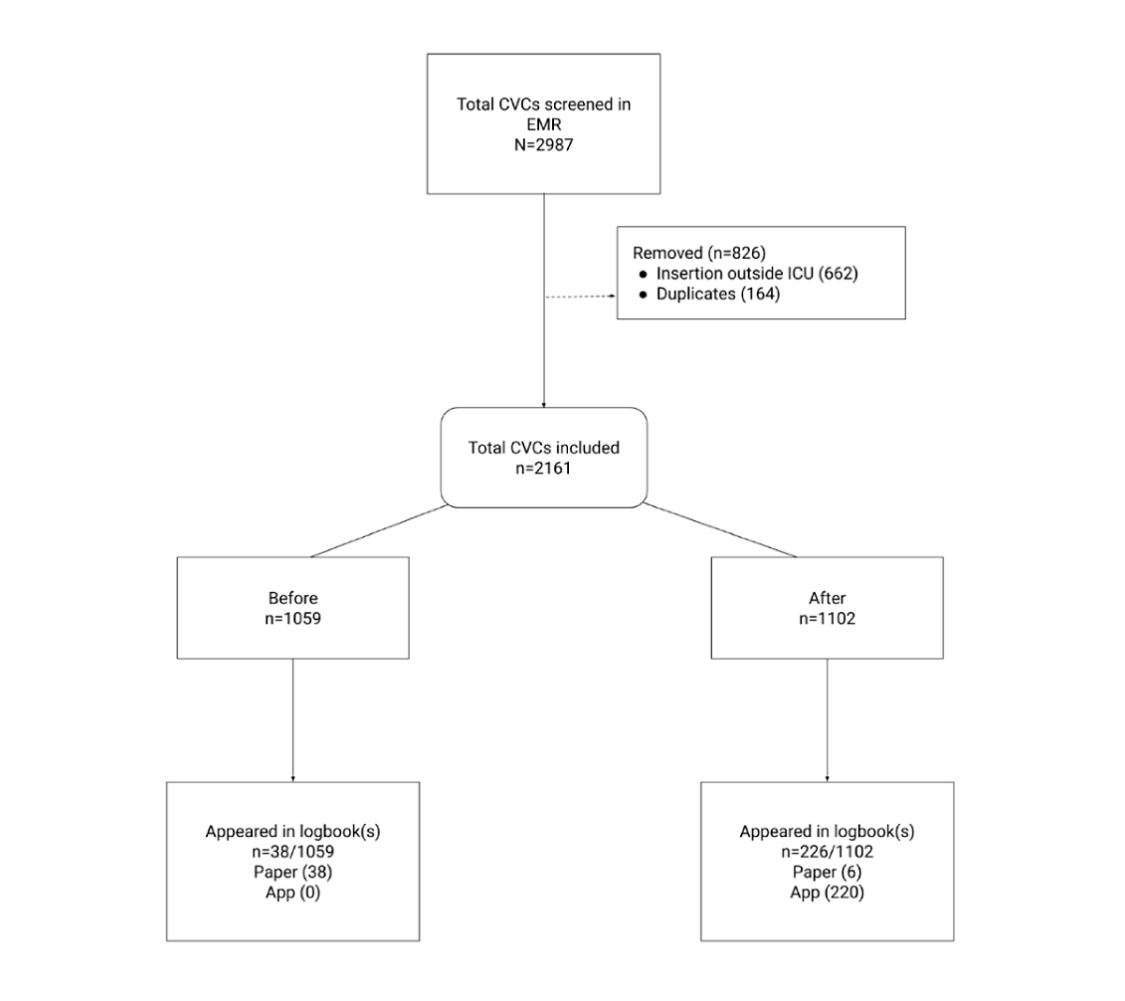
CONSORT diagram. CVC: central venous catheter; EMR: electronic medical record; ICU: intensive care unit.

A total of 2987 CVC insertions recorded in the EMR between April 7, 2019, and April 6, 2021, were screened. Of these, 2161 were included, with 1059 in the before group and 1102 in the after group. There was a total of 270 insertions that had a corresponding logbook entry, 44 in the paper logbook and 226 in the app-based logbook (Figure 1).

The types of lines inserted and their insertion locations were well matched at baseline in the before and after groups (Table 1).

**Table 1:** Central lines recorded in the electronic medical record in patients admitted to the intensive care unit before and after the introduction of the app-based logbook.

Line type and insertion site		Before	After
Total number of lines n		1059	1102
**Standard CVC,^a^ n (%)**			
	External jugular vein	2 (0.2)	6 (0.5)
	Femoral	108 (10.2)	168 (15.2)
	Internal jugular vein	377 (35.6)	394 (35.8)
	Other	0 (0)	3 (0.3)
	Subclavian	169 (16.0)	120 (10.9)
**PICC,^b^ n (%)**			
	Basilic	222 (21.0)	233 (21.1)
	Brachial	54 (5.1)	37 (3.4)
	Cephalic	38 (3.6)	34 (3.1)
**Hemodialysis catheter, n (%)**			
	Femoral	27 (2.5)	26 (2.4)
	Internal jugular vein	54 (5.1)	80 (7.3)
	Other	2 (0.2)	2 (0.2)
	Subclavian	14 (1.3)	5 (0.5)

^a^CVC: central venous catheter.

^b^PICC: peripherally inserted central catheter.

### Study Outcomes

**Figure 2:**
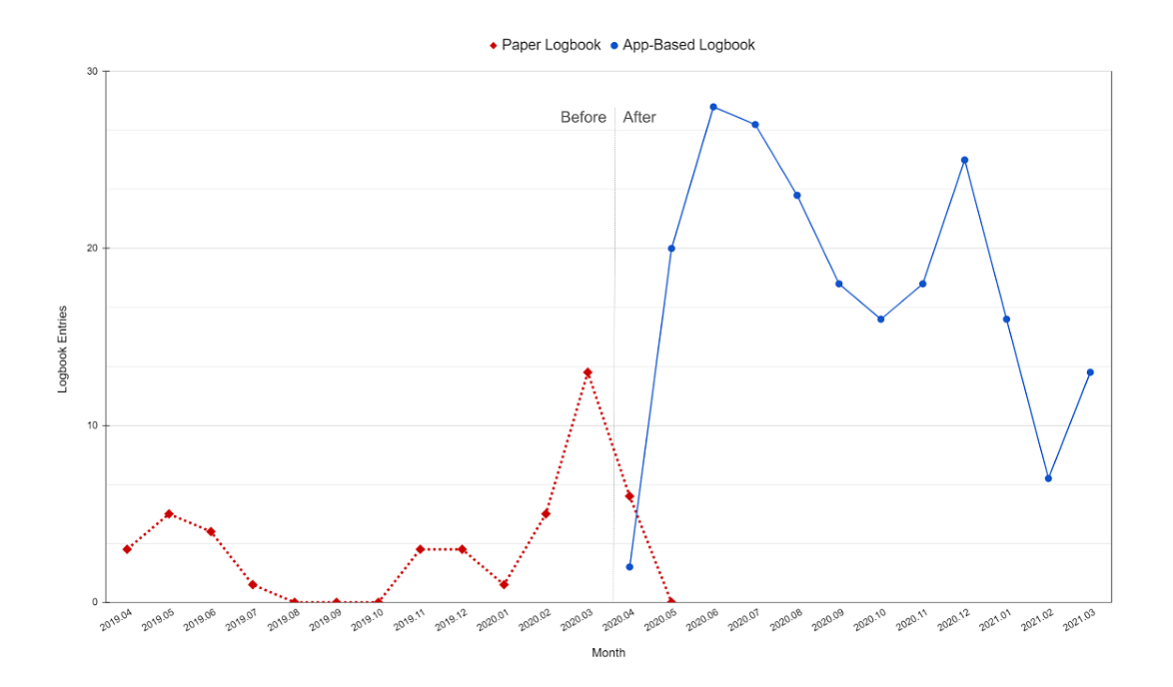
Combined paper-based and app-based central venous catheter logbook entries per month.

The rate of logbook entries increased after the introduction of the app-based logbook (Figure 2).

There was a statistically significant increase in the percentage of logbook entries out of the total number of CVC insertions documented in EMR, from 3.6% (38/1059) in the before group to 20.5% (226/1102) in the after group (*P*<.001; Table 2).

**Table 2:** Percentage of central venous catheter insertions with corresponding logbook entries before and after the introduction of the app-based logbook.

	Before	After	*P* value
Total number of CVCs,^a^ n	1059	1102	N/A^b^
Corresponding logbook entry, n (%)	38 (3.6)	226 (20.5)	<.001
No corresponding logbook entry, n (%)	1021 (96.4)	876 (79.5)	<.001

^a^CVC: central venous catheter.

^b^N/A: not applicable.

There was no difference in the proportion of entries containing a supervisor’s signature from the total number of logbook entries before and after the introduction of the app (*P*=.31; odds ratio 0.65, 95% CI 0.29-1.49; Table 3).

**Table 3:** Percentage of logbook entries before and after the introduction of the app-based logbook containing a valid signature.

	Before	After	Odds ratio	95% CI	*P* value
Total number of logbook entries, n	38	226	N/A^a^	N/A	N/A
Entries with valid supervisor signature, n (%)	29 (76.3)	188 (83.2)	0.65	0.29-1.49	.31

^a^N/A: not applicable.

The number of unique trainees was 146, with 85 counted in the “before” group and 86 in the “after” group. Moreover, 25 trainees were counted in both groups having worked in the John Hunter Hospital ICU during both periods. The training level of the participating trainees in both groups is summarized in Table 4.

**Table 4:** John Hunter Hospital intensive care unit junior doctors by level of training in the before and after time periods.

Trainees	Before, n/N (%)	After, n/N (%)
Senior registrar	4/15 (26)	0/12 (0)
Registrar	14/25 (56)	15/28 (53)
SRMO^a^	39/41 (95)	34/43 (79)
RMO^b^	4/4 (100)	9/10 (90)

^a^SRMO: senior resident medical officer.

^b^RMO: resident medical officer.

There was an overall increase in the rate of active logbook users, from 15.3% (13/85) to 62.8% (54/86) of all trainees. Of these active users, 10 (76.9%) qualified as first-time users in the “before” group and 39 (72.2%) in the “after” group. There was no significant change in adherence between groups with 60% (6/10) first-time users in the “before” group and 79.5% (31/39) in the “after” group going on to log at least 3 or more CVCs during their time working in the unit (*P*=.20; odds ratio 0.39, 95% CI 0.09-1.71; Table 5)

Senior resident medical officers had the greatest increase in the rate of logbook adoption following the introduction of the app-based logbook, going from 5.9% (5/85) to 37.2% (32/86) of all trainees in their first term making at least 1 logbook entry (absolute risk increase 31.3%).

**Table 5:** Adoption and adherence to a logbook. Total number of logbook users, first-time logbook users, and first-time users completing 3 or more entries at John Hunter Hospital intensive care unit.

Level of training		Before	After	Odds ratio	95% CI	*P* value
Unique trainees, n		85	86	N/A^a^	N/A	N/A
**Active users, n (%)**						
	Total	13 (15.3)	54 (62.8)	0.11	0.05-0.22	<.001
	Senior registrar	3 (3.5)	4 (4.7)			
	Registrar	4 (4.7)	10 (11.6)			
	SRMO^b^	5 (5.9)	32 (37.2)			
	RMO^c^	1 (1.2)	8 (9.3)			
**First-time users (3 or more entries) by total first-time users, n/N (%)**						
	Total	6/10 (60)	31/39 (79.5)	0.39	0.09-1.71	.20
	Senior registrar	1/2 (50)	0/0 (0)			
	Registrar	0/2 (0)	6/7 (85.7)			
	SRMO	4/5 (80)	21/24 (87.5)			
	RMO	1/1 (100)	4/8 (50)			

^a^N/A: not applicable.

^b^SRMO: senior resident medical officer.

^c^RMO: resident medical officer.

## Discussion

### Principal Findings

In this retrospective before-and-after study, the addition of a mobile app-based logbook to an existing paper logbook resulted in increased compliance among trainees to the logbook component of a CVC insertion accreditation program without reducing the quality and completeness of logbook entries. This increase occurred without there being an increase in the total number of CVC insertions documented in EMR. There was also an increase in the number of active logbook users while maintaining the same level of adherence.

There are several potential explanations for this observed increase in logbook compliance among our study participants. Smartphones have become ubiquitous in hospitals, with many clinicians using mobile apps as a resource for instant access to information and as clinical decision-making tools [18,20].

We have seen earlier studies show widespread uptake of electronic procedural logbooks following their introduction among trainee doctors [15,21,22]. The addition of a smartphone app makes the digital logbook immediately available for rapid data entry, saving the clinician additional time and energy and allowing them to comply with the completion of their logbook.

There was no difference in the proportion of logbook entries with a valid supervisor signature in the before and after periods, demonstrating that the quality and completeness of the logbook entry as perceived by the supervisors was not reduced, nor was the level of overall supervisor interaction with the logbook process. This means that the addition of a mobile app-based logbook increased the quantity of entries while maintaining quality and supervisor participation.

Junior trainee uptake was higher following the introduction of the app, with the total number of active logbook users during the “before” and “after” periods significantly increasing. This indicates that the increased rate of logbook entries was due to more trainees using the logbook rather than the same number of trainees using the logbook more frequently. It reflects a higher rate of adoption among trainees, which may be due to the convenience and availability of the app, but also due to a generational aspect with most trainees in the study likely to have grown up with digital devices as part of daily life [23]. The increased uptake was more apparent in the senior resident medical officer groups who tended to consist of more junior doctors, and less so in the slightly more senior registrar groups, potentially less conducive to change and acceptance of a new process.

While the rate of new users can give some indication of the app’s performance, it can be confounded by a high “churn” rate; the rate of subscribers, in this case to a logbook, who discontinue use early after their first interaction [24]. We observed an increase in the number of first-time logbook users without compromising the number going on to log multiple entries. Features in the app such as recognition of procedural milestones and automated emails may have contributed to a low churn rate among trainees subscribing to the app-based logbook.

### How Findings Are Different From Previous Knowledge

There are some important differences between previous research and our study. The outcome measures of other studies did not compare relative numbers of CVC insertions logged before and after the introduction of an electronic-based system. Outcome measures in previous studies included absolute numbers of logbook entries, quality of data recorded, and survey-based trainee feedback. Many were conducted using surgical trainees among whom procedural logbooks had been a long established training requirement [15,21]. Although 1 study reviewed logbooks already available as mobile phone apps [18], all others focused on website-based logbooks. Moreover, some studies were conducted before smartphones were widely in use [15,16,21].

### New and Relevant Data the Study Provides

This is the first before-and-after study directly comparing trainee compliance to a procedural logbook in an ICU setting. Although the benefits of electronic logbooks have been studied previously, this is the first time a mobile app has been observed as an intervention.

### Meaning and Implications of the Study

While we have known about the utility of logbooks to facilitate practical aspects of medical training for decades, their application in real world clinical environments has been limited. The inconvenience and time-consuming aspects of accessing and maintaining a physical record is thought to be responsible for the low compliance rate of trainees to their logbooks. This is especially true in a busy ICU environment, where carrying extra documentation or accessing a desktop computer may not be feasible.

This study has shown a significant increase in compliance among trainees in an ICU working toward accreditation in CVC insertion when given the opportunity to use an app compared with a paper logbook, while maintaining the same level of supervision.

Although this was a single-center study, these findings could be generalized to other similar units to ours because the app would be the same everywhere.

### Limitations

As a before-and-after study, the difference between the cohorts may be due to factors other than just the introduction of the logbook app. The introductory sessions for the app may have increased awareness and enthusiasm toward the use of logbooks. There may also be a component of “gizmo idolatry” among junior staff; a conviction that a more technological solution to a problem is intrinsically better than a less technological one [25].

Additionally, the study only measured surrogate outcomes. Patient-centered outcomes, namely CVC related complications, were not directly tested. Trainee and supervisor satisfaction were not addressed.

### Unanswered Questions

A longer-term study is required to reduce the short-term effects of early adoption of a new technology. A multiple unit study is required to test the external validity of this study and for the scalability of such an app-based logbook. Future research is needed to assess an association between logbook compliance and a reduction in CVC-related complications.

### Conclusions

In this before-and-after study, when compared with a previous paper-based system, the addition of a mobile app-based logbook improved the compliance of junior doctors undertaking a CVC insertion accreditation program in an adult intensive care unit, at a tertiary level teaching hospital. There was a marked increase in the number of logbook entries observed as a proportion of all CVC insertions in the unit. Despite this increase, the quality and completeness of entries was not affected. We also observed an increase in the total number of active logbook users after the introduction of the app, with no effect on the average number of logbook entries per user. The more junior trainees accounted for most of this observed increase, potentially signaling a greater willingness to adopt new technologies than their more advanced colleagues. Further studies on app-based logbooks in critical settings are warranted to examine patient outcomes and trainee satisfaction. With digital tools becoming increasingly common in teaching hospitals, it is important to continue to research their impact on medical training and education.

## Abbreviations

CVC: central venous catheter
EMR: electronic medical record
ICU: intensive care unit
